# Influence of functional polymorphism in MIF promoter on sudden cardiac death in Chinese populations

**DOI:** 10.1080/20961790.2017.1327744

**Published:** 2017-05-22

**Authors:** Zhixia Yin, Qing Zhang, Wei Zhou, Shouyu Wang, Chaoqun Wang, Yan He, Lijuan Li, Yuzhen Gao

**Affiliations:** a Department of Forensic Medicine, Medical College of Soochow University, Suzhou, China; b Department of Epidemiology, Medical College of Soochow University, Suzhou, China

**Keywords:** Forensic science, forensic pathology, death, sudden, cardiac, genetic predisposition to disease, macrophage migration inhibitory factor, −794(CATT)_5–8_ polymorphism, genetic susceptibility

## Abstract

Sudden cardiac death (SCD) is defined as an unexpected natural death without any obvious non-cardiac causes that occurs within 1 h with witnessed symptom onset or within 24 h without witnessed symptom onset. Genetic studies conducted during the past decade have markedly illuminated the genetic basis of the cardiac disorders associated with SCD. Macrophage migration inhibitory factor (MIF) is an upstream immunoregulatory cytokine associated with the pathogenesis of many inflammatory diseases including atherosclerosis and myocardial infarction. Previous studies have reported that the functional −794(CATT)_5–8_ polymorphism in MIF is unrelated to sudden infant death syndrome susceptibility. However, there are no reports concerning the association between the polymorphism and adult SCD susceptibility. In the current study, we investigated the association between the −794(CATT)_5–8_ polymorphism and adult SCD susceptibility using 79 adult SCD cases and 313 healthy controls. All samples were analysed using a conventional polymerase chain reaction (PCR) technique. We found that CATT_6_ and 5–6 were the most common allele and genotype in both groups, respectively, while no significant association was found between the −794(CATT)_5–8_ polymorphism and SCD susceptibility. We also summarized the allele frequencies of −794(CATT)_5–8_ in cohorts of healthy people from different countries and found that the allele frequency distributions of the polymorphism in Chinese populations were quite different from that of American and European populations (*P* = 0.005, *P* = 0.0001, respectively), but similar to Japanese populations (*P* = 0.827). In conclusion, this study indicates that the −794(CATT)_5–8_ polymorphism may not be associated with adult SCD susceptibility in Chinese populations. Different allele frequency distributions of the polymorphism in multiple populations may provide a useful reference for further genetic association studies.

## Introduction

In the last decade, sudden cardiac death (SCD) has become a potentially serious public health problem. SCD is defined as an unexpected natural death from a cardiac cause that occurs within 1 h of symptom onset (witnessed) or within 24 h from the last being observed in normal health (unwitnessed) [1]. SCD is considered the major cause of overall sudden death and accounts for at least 10% of natural mortality in the general population [2,3]. The most common cause of SCD is myocardial infarction, especially in individuals 45–50 years of age or older [4]. Molecular epidemiology studies have identified numerous genetic markers related to SCD susceptibility, including genes involved in channelopathies such as Brugada syndrome [5], long QT syndrome [6] and cardiomyopathies, like hypertrophic cardiomyopathy [7], dilated cardiomyopathy [8] and arrhythmogenic right ventricular cardiomyopathy [9]. Although significant progress has been made in revealing the mechanism behind SCD, because of the various causes of SCD and the rarity of subsets of patients under observation or close monitoring, discovering the exact underlying mechanism is challenging. It, therefore, remains an important research topic in both clinical and forensic medicine.

Macrophage migration inhibitory factor (MIF), widely expressed in monocytes, macrophages and T-cells [10], is an upstream immunoregulatory cytokine that contributes to the pathogenesis of many acute and chronic inflammatory diseases such as septic shock [11], rheumatoid arthritis [12] and systemic lupus erythematosus [13]. MIF has emerged as a key factor in cardiovascular diseases [14]. In the cardiovascular system, upregulated MIF was found in both vascular endothelial and smooth muscle cells in atherosclerotic plaques, and was involved in macrophage accumulation and atherosclerotic plaque formation and progression [15]. Upregulation of MIF also contributes to acute myocardial infarction and heart dysfunction [16].

**Figure 1.**
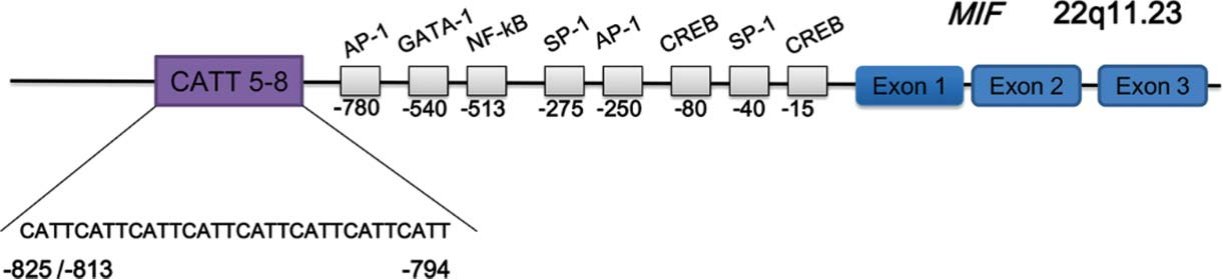
Structure of the human macrophage migration inhibitory factor (MIF) gene. Diagram illustrating three exons of *MIF*, putative transcription factor-binding sites and the −794(CATT)_5–8_ microsatellite repeat.

MIF is located at 22q11.23 and contains several polymorphisms, including the short tandem repeat −794(CATT)_5–8_, which is a microsatellite repetition of CATT at position −794 (Figure 1). It is reported that this repeat regulates basal and stimulus-induced gene transcriptional activity, which increases almost in proportion with repeat number in *in vitro* assay systems. Reporter gene assays have demonstrated that the CATT_5_ allele has the lowest basal level and stimulated MIF transcriptional activity compared with the CATT_6_ and CATT_7_ alleles [17,18]. Recently, Yao et al. [19] reported that ICBP90 (also known as UHRF1) was the major transcription factor interacting with the MIF microsatellite and exerted a central influence on MIF expression. Studies have reported that the functional −794(CATT)_5–8_ polymorphism in MIF was unrelated to sudden infant death syndrome (SIDS) susceptibility [20]. However, there are no reports concerning the association between polymorphism and adult SCD susceptibility.

The current study was a case-control study aimed at analysing the association between −794(CATT)_5–8_ polymorphism and adult SCD susceptibility in Chinese populations.

## Materials and methods

### Recruitment criteria

The study is an independent case-control set including 79 SCD cases and 313 controls. All cases were genetically unrelated and of ethnic Han Chinese descent. Blood samples of SCD cases were recruited from Medicolegal Expertise Center of Sun Yat-sen University, Medicolegal Expertise Center of Xiangya Medical University, Institute of Forensic Science, Ministry of Justice and Soochow University between 2012 and 2016. Exhaustive toxicological examinations were performed in all cases to exclude the possibility of poisoning death. The 79 SCD cases were assumed to suffer sudden death caused by coronary heart disease since no lethal pathological features but varying degrees of coronary atherosclerosis were discovered. A total of 313 healthy controls without any cardiovascular disease history or sudden death family history were frequency matched for age (±5 years) and gender to SCD cases recruited from the community nutritional survey conducted in the same regions during the same period as the victims.

### DNA extraction and genotyping

A genomic DNA purification kit (Qiagen, Germantown, MD, USA) was used to extract genomic DNA from blood samples. DNA fragments containing −794(CATT)_5–8_ were amplified using a pair of genotyping primers (forward primer: 5′-ACCTGGCCTGTGATCCAGTT-3′, reverse primer: 5′-AGGTGCCAGGCATACAAGAGA-3′) synthesized by Genewiz Company (Suzhou, China). Polymerase chain reaction (PCR) products were analysed using 7% non-denaturing polyacrylamide gel electrophoresis and visualized using silver staining [21]. Genotyping was conducted in a double-blind manner as described previously [22]. Quality control was performed by way of direct sequencing of 20 randomly selected DNA samples to validate the genotyping method. Approximately 10% of the total samples randomly selected were examined in duplicate by two independent technicians to confirm 100% consistency.

### Statistical analysis

Genotype distribution in the control group was analysed using Hardy–Weinberg equilibrium and the chi-squared test. Logistic regression was used to assess the associations between −794(CATT)_5–8_ and SCD susceptibility, adjusted by gender and age. Allele frequency differences among populations were examined using the Fisher's exact test. Statistical analyses were conducted using SPSS software (IBM Corp. Released 2010; IBM SPSS Statistics for Windows, ver. 19.0. Armonk, NY, USA). A *P-*value <0.05 was considered statistically significant. All statistical tests were two-sided.

## Results

### Associations between −794(CATT)_5–8_ polymorphism and SCD susceptibility

The demographic characteristics of the SCD cases and matching controls are summarized in Table 1. The median ages of SCD cases and controls were 50 and 49 years, respectively. There was a significant difference in the frequency of gender, 91.1% were male and 8.9% were female. The characteristics of the 79 SCD cases are listed in Table S1. The majority of the deceased (53.2%) suffered death after falling, following slight injuries (defined as nonspecific); 2 cases (2.5%) happened after intense exercise or heavy physical activities, 11 cases (13.9%) occurred while sleeping, and 24 cases (30.4%) occurred during quarrels or other strongly emotional activities.

**Table 1.** Clinical characteristics of SCD cases and controls.

Characteristic	SCD (*N* = 79)	SCD matched controls (*N* = 313)
Sex, No.		
Male	72	281
Female	7	32
Age, years old, mean ± SD (range)		
Overall	49.87 ± 13.58 (19–79)	49.12 ± 10.15 (25–78)
Males	48.96 ± 12.41 (19–77)	48.75 ± 10.05 (25–78)
Females	59.29 ± 21.48 (27–79)	60.77 ± 12.54 (38–70)
Events at sudden death, No.		
Sleep	11	
Nonspecific	42	
Exertion	2	
Stress	24	
Symptoms before sudden death, No.		
None	52	
Others	27	
Megalothymus, No.		
Positive	2	
Negative	77	

SCD: Sudden cardiac death

**Figure 2.**
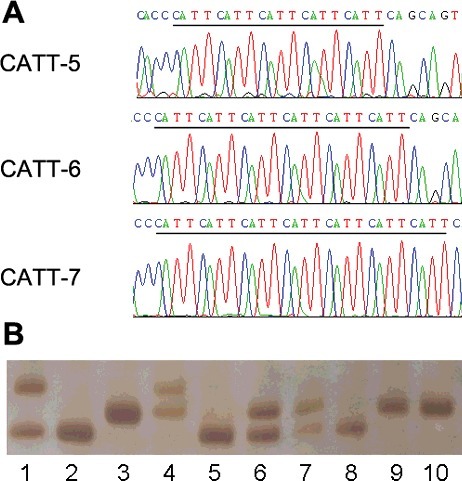
Example output from sequencing and genotyping assays of the **−**794(CATT)_5–8_ polymorphism. (A) Sequencing results of the −794(CATT)_5–8_ polymorphism in template strands. Underlined bases indicate the CATT repeat number. (B) Genotyping outcomes using 7% non-denaturing polyacrylamide gel electrophoresis (PAGE) and silver staining (lane 1: 5–7 genotype; lane 2, 5, 8: 5–5 genotype; lane 3, 9, 10: 6–6 genotype; lane 4: 6–7 genotype; lane 6, 7: 5–6 genotype).

Examples of genotyping assays and sequencing results for −794(CATT)_5–8_ are presented in Figure 2. The genotypic frequencies of −794(CATT)_5–8_ observed in the control group conformed to Hardy–Weinberg equilibrium (*P* > 0.05). Genotype and allele frequencies of −794(CATT)_5–8_ and odds ratios with 95% confidence intervals are presented in Table 2.

**Table 2.** Associations between −794(CATT)_5__–__8_ and SCD susceptibility.

	Genotype						Allele		
Group	5–5	5–6	5–7	6–6	6–7	7–7	5	6	7
SCD (%)	12 (15.19)	33 (41.77)	6 (7.60)	10 (12.66)	16 (20.25)	2 (2.53)	63 (39.87)	69 (43.67)	26 (16.46)
Control (%)	47 (15.02)	110 (35.14)	36 (11.50)	66 (21.09)	43 (13.74)	11 (3.51)	240 (38.34)	285 (45.53)	101 (16.13)
OR (95% CI)^a^	0.85 (0.38–1.89)	1.00 (Reference)	0.56 (0.19–1.54)^b^	0.51 (0.22–1.15)	1.24 (0.58–2.62)	0.61 (0.09–3.13)^b^	1.08 (0.73–1.62)	1.00 (Reference)	1.06 (0.62–1.81)
*P* value	0.671		0.219	0.079	0.542	0.524	0.678		0.812

CI: Confidence interval; OR: Odds ratio.

a: Adjusted for age and gender factors.

b: Fisher's exact test.

When analysing the genotype and allele frequencies for this polymorphism, we observed that the 5–6 genotype was the most frequent in each subgroup (SCD cases: 41.77% and control: 35.14%); nevertheless, we did not find any association between the variant and SCD susceptibility.

### Analysis of allele frequencies of different countries

Table 3 is a summary of allele frequencies of −794(CATT)_5–8_ polymorphism in cohorts of healthy people from different countries. Compared with previous data, we observed a higher frequency of CATT_5_ and CATT_7_ in controls (38.34% vs. 16.13%) compared with that of Germany (31.9% vs. 9.4%). In our case-control set, we did not observe the CATT_8_ allele reported in low frequency (0.6% in America and 0.3% in the UK). The CATT_6_ allele was predominant in people from America, the UK and Spain (60.7%, 65.6% and 65.0%, respectively), whereas CATT_5_ and CATT_7_ alleles were more frequent in Japanese and Chinese people.

**Table 3.** Allele frequencies of −794(CATT)_5__–__8_ in healthy subjects from different countries.

		−794(CATT)				
Population	Subjects	5	6	7	8	Reference
American	159	27.7	60.7	11	0.6	[17]
German	109	31.9	58.8	9.4	0	[20]
Japanese	155	39.4	42.6	17.4	0.6	[26]
UK	342	25.3	65.6	8.8	0.3	[30]
Spanish	886	27.0	65.0	8.0	0	[31]
Mexican	210	19.3	58.6	22.1	0	[32]
Chinese(present study)	313	38.4	45.5	16.1	0	

Table 4 shows the results of the Fisher's exact test for all possible pairwise comparisons. The allele frequency distribution in Chinese was different to that of all the listed countries except people from Japan and Mexico, and there was no significant difference in people from America, the UK, Spain and Germany.

**Table 4.** Comparisons of allele frequencies in different countries.

Population	America	UK	Spain	German	Japan	Mexico	China
America		0.614	0.470	0.607	0.004	0.009	0.005
UK			0.819	0.111	0.0001	0.0001	0.0001
Spain				0.073	0.0001	0.0001	0.0001
German					0.0001	0.0001	0.0001
Japan						0.0001	0.827
Mexico							0.0001
China							

### Analysis of MIF polymorphisms in different diseases

Previous studies have revealed the role of MIF in the pathogenesis of numerous diseases. The relationship between MIF polymorphisms and some diseases according to several articles are summarized in Table 5. The CATT_5_ allele could reduce the disease severity of rheumatoid arthritis in American populations; the 6–7 genotype was associated with acute coronary syndrome in Mexican populations; the CATT_7_ allele increased the severity of carotid artery atherosclerosis; and the CATT_7_–173*C haplotype increased systemic lupus erythematosus susceptibility.

**Table 5.** Association between MIF polymorphisms and different diseases.

Type of disease	Country	Relationship between MIF polymorphisms and disease	Reference
Rheumatoid arthritis (RA)	America	CATT_5_ allele reduced disease severity	[12]
Systemic lupus erythematosus (SLE)	Spain	CATT_7_–173*C haplotype increased susceptibility to SLE	[13]
Acute coronary syndrome (ACS)	Mexico	6–7 genotype increased susceptibility to ACS	[27]
Carotid artery atherosclerosis (CAA)	China(Taiwan)	CATT_7_ allele increased severity of CAA	[28]
Atopy	Japan	CATT_7_–173*C haplotype increased risk to atopy	[26]
		CATT_5_–173*G haplotype reduced risk to atopy	

## Discussion

To our knowledge, this is the first case-control study on the impact of MIF promoter variation on SCD susceptibility in Chinese populations. Previously, a German study explored the influence of the −794(CATT)_5–8_ polymorphism on SIDS and found that this polymorphism was not involved with SIDS [20]. Different to SCD, SIDS is defined as the sudden and unexpected death of an apparently healthy infant younger than one year of age [23], and both respiratory failure and impaired thermoregulation make significant contributions to SIDS [24]. Because of the different pathogeneses between SIDS and SCD, we investigated the association between the −794(CATT)_5–8_ polymorphism and adult SCD susceptibility. However, no significant differences regarding allele or genotype frequencies between SCD and controls were observed, which was consistent with the German study.

It is generally believed that MIF functions as a cytokine to promote the recruitment and migration of neutrophils and macrophages to the site of inflammation [25]. Several studies have shown that MIF is a key modulator in many acute and chronic inflammatory diseases including septic shock [11], rheumatoid arthritis [12], systemic lupus erythematosus [13] and atopy [26]. There are also studies that have investigated the relationship between MIF and cardiovascular disease. For instance, the 6–7 genotype of −794(CATT)_5–8_ polymorphism was found to be associated with an increased susceptibility to acute coronary syndrome in Mexican populations [27]; the CATT_7_ allele could increase the severity of carotid artery atherosclerosis in Chinese populations [28]; and White et al. demonstrated a pro-inflammatory role for MIF in acute myocardial infarction [29]. All these cardiac diseases are closely related to SCD. Nevertheless, no relationship has been found between MIF and SCD. A plausible interpretation could be that MIF may not be a crucial factor, and may have to cooperate with other risk factors to play a role in the pathogenesis of SCD.

We found that the allele frequency in Chinese populations was similar to that of the Japanese populations [26] but different to that of American or European populations [30,31]. The Mexican [32] population included Amerindian, European and African populations, and its allele frequency was not only different to American and European populations but also to Chinese and Japanese populations. The difference in allele frequency distribution may be attributed to human evolution and the ethnic differences stemming from environmental or genetic factors. Furthermore, the sample size and the inclusion criteria in each study could affect allele frequency distributions.

This study had some limitations. The sample size of SCD cases is small. Further case-control studies with larger sample sizes are needed to confirm the representativeness and repeatability of our study. Furthermore, we analysed only one polymorphism, it would be useful to analyse other functional genetic variations of MIF to fully elucidate its functions in the pathogenesis of SCD.

In summary, the data presented here provide initial evidence that the functional polymorphism −794(CATT)_5–8_ may not be associated with adult SCD susceptibility in Chinese populations. Our analysis concerning the different allele frequency distributions of the polymorphism in multiple populations may provide a useful reference for further genetic association studies.

## Compliance with ethical standards

This study was approved by the Ethical Committee of Soochow University. Written informed consent was obtained from relatives of each sudden death case before the investigation.
